# HIV-associated bladder cancer: a case series evaluating difficulties in diagnosis and management

**DOI:** 10.1186/1471-2490-9-10

**Published:** 2009-08-31

**Authors:** Elizabeth M Gaughan, Bruce J Dezube, Mark Bower, David M Aboulafia, Gerry Bohac, Timothy P Cooley, Liron Pantanowitz

**Affiliations:** 1 Department of Medicine (Hematology-Oncology), Beth Israel Deaconess Medical Center, Harvard Medical School, Boston, MA, USA; 2 Department of Oncology, Chelsea and Westminster Hospital, London, UK; 3 Division of Hematology and Oncology, Virginia Mason Clinic and the Division of Hematology, University of Washington, Seattle, WA; 4 Department of Medicine, Rush University, Chicago, IL, USA; 5 Department of Medicine, Lahey Clinic, Burlington, MA, USA; 6 Department of Pathology, Baystate Medical Center, Tufts University School of Medicine, Springfield, MA, USA

## Abstract

**Background:**

Chronic human immunodeficiency virus (HIV) infection is associated with an increased incidence of Non-Acquired Immunodeficiency Syndrome (non-AIDS) defining cancers. To date, only a limited number of cases of bladder cancer have been linked with HIV infection. We sought to describe the clinical characteristics of HIV-associated bladder cancer.

**Methods:**

A retrospective study was performed involving HIV-positive patients with bladder cancer, combining cases from multiple institutions with published case reports. Data regarding patient demographics, HIV status, clinical presentation, pathology, cancer treatment, and outcome were analyzed using descriptive statistics.

**Results:**

Eleven patients were identified with a median age of 55 years (range, 33 - 67). The median CD4+ count at cancer diagnosis was 280 cells/mm^3 ^(range, 106 - 572 cells/mm^3^). Six patients (55%) had a known risk factor for bladder cancer, and nine (82%) presented with hematuria. Ten patients had transitional cell carcinoma, and most had superficial disease at presentation. Treatment included mainly transurethral resection of bladder tumor followed by a combination of local and systemic therapies. One patient received intravesical bacillus Calmette-Guèrin (BCG) without complication. Several patients (55%) were alive following therapy, although many (64%) suffered from local relapse and metastatic disease.

**Conclusion:**

Bladder cancer is part of the growing list of cancers that may be encountered in patients living longer with chronic HIV-infection. Our patients presented at a younger age and with only mild immunosuppression, however, they experienced an expected course for their bladder cancer. Hematuria in an HIV-infected patient warrants a complete evaluation.

## Background

Human immunodeficiency virus (HIV) infection has become a chronic illness for many patients since the advent of highly active antiretroviral therapy (HAART). As their life expectancy increases, patients with chronic HIV infection are developing diseases associated with aging and longer-term exposure to several oncogenic risk factors (e.g. oncovirus coinfection). Specifically, epidemiologic studies in the HAART era have demonstrated an increased incidence of Non-AIDS Defining Cancers (NADC) including lung cancer, skin cancer (other than Kaposi sarcoma), anal cancer, hepatocellular carcinoma, conjunctival cancer, Hodgkin lymphoma, plasma cell neoplasia and leiomyosarcoma in HIV-infected patients [1]. Several factors likely contribute to the development of NADC in this population such as family history, exposure to oncoviruses including human papilloma virus (HPV) and Epstein-Barr Virus (EBV), and tobacco consumption (chewing or smoking). However, HIV-mediated immunosuppression, per se, appears to play a lesser role in carcinogenesis than has been reported with classic AIDS-defining tumors (Kaposi sarcoma, non-Hodgkin lymphoma, and cervical cancer). While HIV infection may not lead to an increased incidence of all malignancies, it can alter the presentation, natural history and potential therapeutic options available to patients afflicted with these neoplasms.

Bladder cancer has been reported in association with HIV infection in a limited number (six) of published case reports [2-6]. Large epidemiologic studies conflict on whether there is an increased incidence of bladder cancer in the HIV-infected population. In a study from Zambia (1980-1989), involving 7836 cases of neoplasia, bladder carcinoma (6.3%) was the third leading malignancy reported, after carcinoma of the cervix (19.6%) and Kaposi's sarcoma (7%) [7]. However, a review of the cancer registry of Uganda for incidence rates from 1989 to 1991 showed a decline in the incidence of bladder cancer with the emergence of AIDS-related Kaposi sarcoma [8]. Linked population-based AIDS and cancer registry data (1978-1996) from the United States, including 302,834 adults with HIV/AIDS, identified 48 people with HIV-associated bladder cancer with a relative risk of approximately 0.6 in this group compared to the general population [9]. A 2007 meta-analysis of several studies involving 444,172 individuals with HIV/AIDS did not demonstrate an increased incidence of bladder cancer in the HIV population compared to the general population [10]. Most recently, a 2008 epidemiologic study using HIV/AIDS Cancer Match Study data reported a lower incidence of bladder cancer in the HIV population as compared to the general population with a standardized incidence ratio of 0.7 [11].

To date, there have been no published series that specifically address the association and management issues related to bladder cancer in the setting of HIV-infection. Therefore, the aim of this study was to investigate a series of HIV-associated bladder cancer cases, together with cases previously published in the literature, and to identify particular risk factors, clinical findings, pathologic features, management issues, and/or response to therapy in this group of immunocompromised patients.

## Methods

Following institutional review board approval, we performed a retrospective, multi-institutional study involving HIV positive patients with concomitant bladder cancer. Cases were identified from the personal archives of the authors for patients with a combined diagnosis of HIV infection and confirmed bladder carcinoma. The literature was searched for additional cases using PubMed and previous article reference lists for all published cases in the English and Non-English literature. We extracted data from the medical records and published papers which included patient demographics (age, gender), HIV status (CD4+ cell count, HIV viral load), clinical presentation, pathology (carcinoma histological subtype and grade according to WHO), cancer treatment, and outcome. Accrued data were analyzed using descriptive statistics.

## Results

We identified 11 patients with confirmed HIV-associated bladder carcinoma; 5 from the authors' personal archives (labeled case 1 through to 5) over a 5-year period (2001-2006) and 6 published reports (1992-2002) from Spain, Italy, Germany, and Qatar [2-6]. The details of this patient cohort are summarized in Table 1.

**Table 1:** Characteristics of patients with HIV-associated bladder cancer.

**Case**	**Age** **(years)**	**Gender**	**CD4 count (cells/mm^3^)**	**HIV load (copies/mL)**	**HAART**	**Clinical Presentation**	**Pathology**	**TNM (stage)** **at diagnosis**	**Therapy**	**Outcome**
Al Soub^2^ 1992	49	Male	270	NR	AZT only*	Frequency, Dysuria, LAP	TCC LG	T1N0M0 (stage I)	Radiation TURBT	Alive 1 year later

Clemente Ramos et al^3^ 1998	33	Male	106	NR	AZT only	Frequency, Dysuria, Hematuria Weight loss	TCC HG	NR	Surgery Systemic chemotherapy	Died 5 months after diagnosis

Wolf^4^ 2001	37	Female	318	NR	No	Hematuria, LAP	TCC HG	T1N0M0 (stage I)	TURBT Radiation	Alive

Santos et al^5 ^2002	51	Male	228	NR	NR	NR	SCC	NR	NR	Alive 4 months later

Manfredi et al^6^ 2000	58	Male	572	1,600	Yes	Hematuria	TCC LG	TaN0M0 (stage 0a)	TURBT	Relapsed with local disease, Alive

Manfredi et al^6^ 2002	57	Male	447	665,000	Yes	Hematuria	TCC HG	T1N0M0 (stage I)	TURBT Local chemotherapy Cystectomy	Relapsed with local disease, Alive 8 months later

Case 1	61	Male	228	<50	Yes	Hematuria	TCC HG Papillary	T1N0M0 (stage I)	TURBT Local chemotherapy Palliative surgery and radiation	Relapsed with metastatic disease, Died 7 months later

Case 2	49	Male	280	<50	Yes	Frequency, Urgency, Dysuria, Hematuria	TCC HG	TaN0M0 (stage 0a)	TURBT Local chemotherapy Cystectomy	Multiple local relapses, Alive 1 year later

Case 3	63	Male	317	50,000	Yes	Hematuria	TCC HG	T3N2M1 (stage IV)	TURBT Systemic chemotherapy	Died within 6 months

Case 4	55	Male	280	<50	Yes*	Hematuria	TCC HG	T1N0M0 (stage I)	TURBT Cystectomy Radiation Craniotomy	Relapsed with metastatic disease, Died 3 years after diagnosis

Case 5	67	Female	445	<50	Yes	Hematuria, LAP	TCC HG	T4N0M0 (stage IV)	Palliative radiation Embolization of one vesical artery	NR

NR = Not reported; LAP = lower abdominal pain; TCC = Transitional cell carcinoma; SCC = Squamous cell carcinoma; HG = High grade; LG = Low grade; AZT = zidovudine; * = started after bladder cancer diagnosis; HAART = highly active antiretroviral therapy; TURBT = transurethral resection of bladder tumor

### Clinical Findings

The median patient age was 55 years (range, 33 - 67 years), and the majority were male (M:F 9:2). Five patients were Caucasian and one was Hispanic. The race of the other six patients was not reported. Ten patients (91%) had known HIV infection at the time of their bladder cancer diagnosis, and the eleventh patient was diagnosed with HIV and bladder cancer concurrently. The median CD4+ cell count was 280 cells/mm^3 ^(range, 106 - 572 cells/mm^3^). An HIV viral load was available in seven of the cases; mean HIV viral load was 102,400 copies/ml (range, <50 to 665,000 copies/mL). The mean reported duration of HIV infection prior to the development of bladder cancer was 8 years (range, 1-25 years). A total of six (55%) patients were on HAART at the time of diagnosis of their bladder malignancy and one initiated HAART upon starting cancer treatment. Two of the patients were on trimethoprim-sulfamethoxazole prophylaxis. Compliance with taking antiretroviral medication and HIV resistance data were not available for most cases, but two patients had known poor compliance with HAART therapy. One patient (case 1) had stopped his antiretrovirals for three months prior to the incidental presentation with microscopic hematuria on routine urinalysis. Two case reports from the literature described patients diagnosed with bladder cancer prior to the advent of HAART therapy [2,3]. Both of these patients were treated with zidovudine monotherapy around the time of their cancer treatment.

In general, this cohort did not suffer from multiple medical co-morbidities or opportunistic infections. A single patient had a history of both Kaposi sarcoma and oral candidiasis. Three patients had hepatitis C co-infection and one had both hepatitis B and hepatitis C co-infection. Two patients were actively using intravenous drugs and two patients had hypertension. One patient had a history of cervical cancer treated with radiation therapy, which developed decades prior to HIV and bladder cancer diagnoses.

Six (55%) patients had at least one known risk factor for the development of bladder cancer. Five (45%) had a history of smoking, and one patient had previous pelvic radiation therapy. Two patients suffered from recurrent cystitis, and three had a history of genitourinary calculi. One patient had recurrent urinary tract infections as a result of radiation cystitis and small bladder volume from pelvic radiation. Another patient had recurrent urinary tract infections associated with a history of bladder stones (including indinavir-related stones). There were no reported occupational or other chemical exposures in this group of patients. Also, no patient had a reported history of significant analgesic use, exposure to chemotherapeutic agents (i.e. cyclophosphamide) or a family history of bladder cancer.

All ten patients with a reported clinical presentation had signs or symptoms which led to the diagnosis of bladder cancer. Nine (82%) patients presented with hematuria, and in five cases this was the only presenting sign. Microscopic hematuria was identified in four patients, incidentally in two cases, and during evaluation for urinary symptoms in another two cases. Five patients presented with or developed gross hematuria with blood clots, including two patients who initially had microscopic hematuria. Three patients (27%) had lower abdominal pain on presentation and four (36%) complained of irritative symptoms such as frequency, urgency and/or dysuria. One individual had notable weight loss and was found on physical examination to have a large mass invading the anterior vaginal wall.

**Figure 1:**
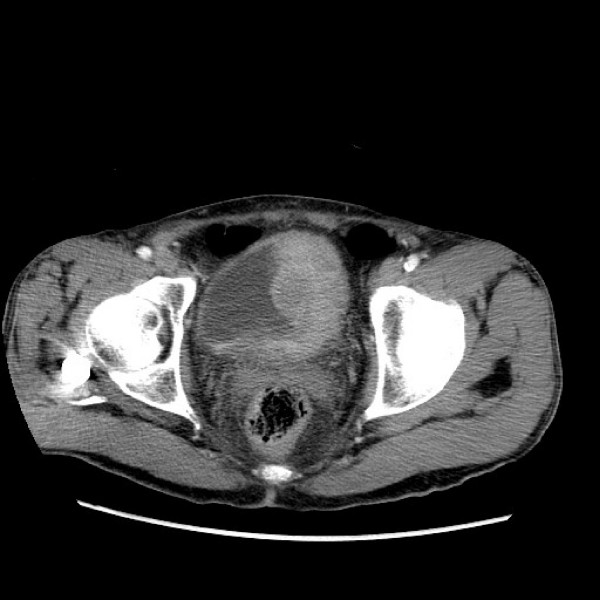
**CT scan of Case 1**. large lobulated, enhancing soft tissue mass affecting the left side of the bladder (a).

**Figure 2:**
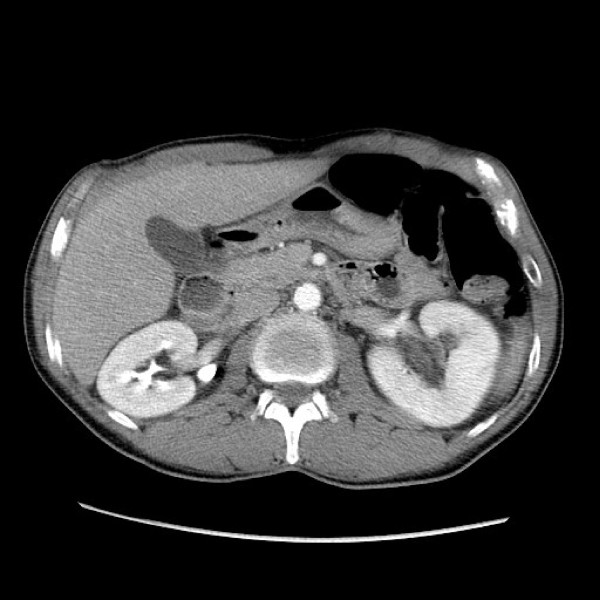
**CT scan of Case 1: bladder cancer mass with associated left hydronephrosis and hydroureter**.

The radiologic and urologic evaluation of the urinary symptoms involved several modalities including urine cytology, ultrasound, intravenous pyelogram (IVP), computerized tomography (CT) scans and cystoscopy. The diagnostic tests used in these patients varied based on available technology at the time of diagnosis and degree of symptoms. Three patients had urine cytology for initial evaluation, and only one had a positive result. Two patients underwent IVP, and one study was positive for a large filling defect and a bladder calculus, and the other did not demonstrate any abnormalities. Ultrasound of the urinary system was the initial test in three patients, including two patients with incidental microscopic hematuria. This modality suggested a muscle-invading bladder tumor in one patient, bilateral nephrolithiasis in one patient and was normal in the third patient. CT scans, including the CT-urogram technique, was used in seven patients for evaluation of symptoms and/or for staging [2,3,6]. Bladder wall thickening and hydronephrosis were the most common abnormalities identified. Three patients were found to have locally invasive or metastatic disease by CT scan (Figures 1 and 2).

Ultimately, all ten patients underwent cystoscopy for tissue diagnosis and local tumor management. Most patients had papillary masses identified, but one patient had a sessile tumor involving most of his bladder wall [3]. Three patients had grossly necrotic tissue visible and one patient had tumor completely filling the bladder. The patient with a history of pelvic radiation was found to have a necrotic, calcified posterior bladder wall, and random biopsies demonstrated bladder cancer. Two patients had multifocal papillary tumors on initial cystoscopy, one with a papillary tumor visible in the prostatic urethra. Two patients had stones (seen previously on imaging) in the bladder [2].

### Pathologic Features

**Figure 3:**
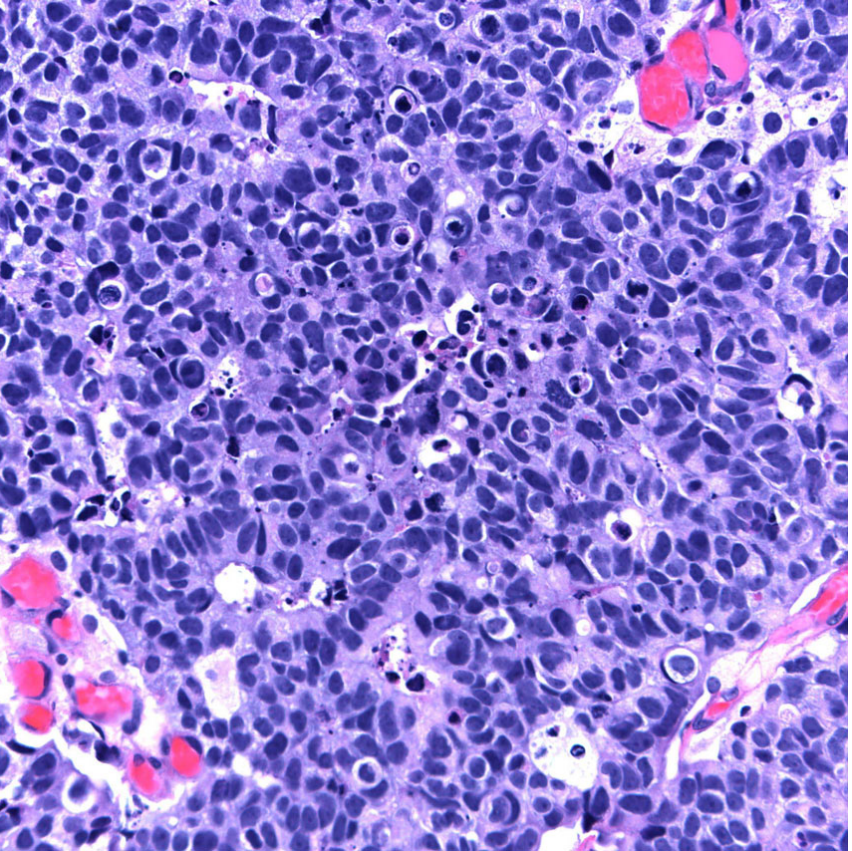
**invasive high grade transitional cell carcinoma with neuroendocrine features (H&E stain)**.

**Figure 4:**
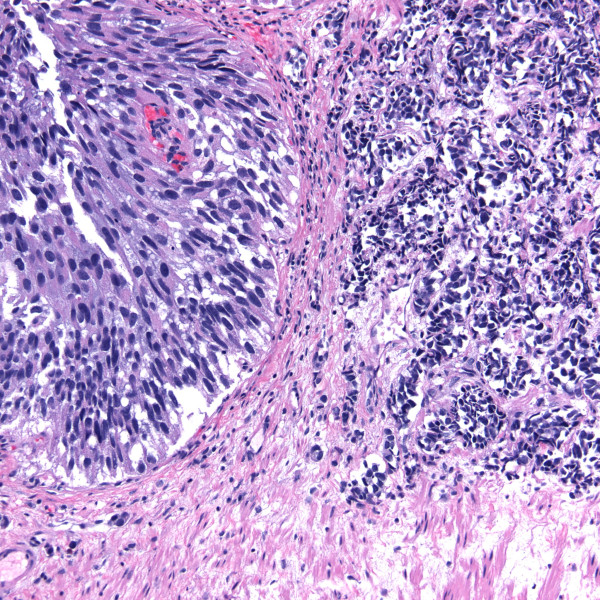
**invasion of the urinary bladder muscularis propria and local lymphatic vessels (H&E stain)**.

Most cases (10/11) had transitional cell carcinoma (TCC; also referred to as urothelial carcinoma) that was high grade in eight and low grade in two individuals. In four of these cases a papillary architecture (i.e. papillary TCC) was reported. Three patients had necrotic tumors at presentation. One patient (case 1) with recurrent tumors, had neuroendocrine features identified on pathology (Figure 3). There was only one case of squamous cell carcinoma (SCC). There was sufficient data for TNM staging at the time of bladder cancer diagnosis in 9 cases. The tumor was non-invasive (Ta) in two patients, invaded the subepithelial urinary bladder connective tissue in five (56%) cases (Figure 4), and the perivesical tissue (T3) in one individual. One person had stage T4 disease as a result of tumor invasion into the vaginal wall. Only one patient had identifiable lymph node (N2) involvement and concomitant distant (M1) metastases at initial presentation. At the time of bladder cancer presentation, two patients had stage Ta disease, five had stage I disease, and two had stage IV disease. Three patients had metastatic disease during their course and the sites of involvement included bone, brain and regional lymph nodes. In one case (case 2), following radial cystoprostatectomy prostatic adenocarcinoma of Gleason grade 3 + 3 = 6/10 was discovered incidentally involving approximately 5% of prostatic volume. In another case, prostatic intraepithelial neoplasia was identified.

### Patient Management

Information regarding treatment was available in 10 cases. Transurethral resection of bladder tumor (TURBT) was performed in eight cases, including all cases of superficial disease. Five patients required repeat TURBT because of relapsed disease. Three patients with T1 disease were given intravesical chemotherapy following surgical resection. Each received at least one cycle of mitomycin C, with one patient receiving five cycles of this therapy. One patient was given a cycle of intravesical epirubicin and another was given intravesical bacillus Calmette-Guèrin (BCG), both following relapse after mitomycin C therapy. There were no infectious complications after the BCG infusion. All patients treated with intravesical therapy relapsed and ultimately required cystectomy. One patient with T1 disease underwent radical cystectomy following his initial TURBT, instead of local therapy. Radical cystectomy or cystoprostatectomy was attempted in four patients, and three patients had an orthotopic neobladder created. One of these individuals was found to have tumor adherent to the pelvic side wall and the procedure was aborted. Two patients had external beam pelvic radiation therapy for treatment of their bladder tumors. The first received 5000 Gy over 28 days and did not respond [2]. The second received 60 Gy complicated by radiation cystitis, genital inflammation and diarrhea, but was successful at controlling the disease [3]. Three patients received palliative radiation to the bladder and sites of metastatic disease (brain and bone), complicated by hemorrhagic cystitis in two patients. One patient underwent vesical artery embolization for palliation of recurrent gross hematuria. Two patients were treated with systemic therapy, including methotrexate/vinblastine/doxorubicin/cisplatin (MVAC), gemcitabine/cisplatin, and gemcitabine/carboplatin. Both of these patients developed renal impairment and ultimately died from advanced and progressive bladder cancer.

### Patient Outcome

Several of the patients (6/11; 55%) were alive within four months to one year after their case was reported. However, five (71%) of these individuals relapsed with local disease (3 cases), in case 2 multiple times, or with metastases (2 cases). Relapse in one patient (case 1) manifested with symptoms mimicking pyelonephritis (acute costovertebral angle tenderness and infection on urinalysis). However, a CT-urogram in this individual demonstrated a large lobulated, enhancing soft tissue mass affecting the left side of the bladder, and associated left hydronephrosis and hydroureter (Figure 2). Subsequent cystoscopy identified a large volume of tumor, predominantly involving the left lateral bladder wall that extended anteriorly and across the trigone to the right side. A left obturator lymph node excision in this patient showed metastatic carcinoma and one month later he presented with diffuse vertebral metastases. Case 2 was also hospitalized after cystoprostatectomy for right ureteral colic and pyelonephritis and ultimately had a right nephrostomy tube placed along with lithotripsy for recurrent stone disease. The two patients with Ta tumors each initially underwent TURBT, but both ultimately relapsed with superficial disease. Of the patients who had or developed T1 disease, two survived without further disease, two relapsed with superficial tumors and required cystectomy but survived, and two relapsed with metastatic disease and died. Overall, four (36%) of these patients died, 3 as a result of their bladder carcinoma and one (case 1) due to aspergillus pneumonia. All patients died within 5-7 months of their diagnosis of metastatic or locally invasive disease, except for one person (case 4) who died 3 years after his initial cancer diagnosis.

## Discussion

In this study, we present the first series of HIV-associated bladder cancer consisting of 11 patients compiled from our case files and case reports from the literature. Based upon only single case reports it has been difficult to determine if this NADC is associated with particular risk factors, clinical manifestations, pathology, biological behavior, or management issues in the setting of HIV infection. While studies to date have not been able to demonstrate an increased incidence of bladder cancer in the HIV population, we identified certain clinical factors in this group of immunocompromised individuals which may affect the diagnosis and management of bladder cancer [5].

There are approximately 68,000 new cases of bladder cancer diagnosed each year in the United States, with 75% of cases occurring in men [12]. Our male-to-female ratio was consistent with this trend. Bladder cancer is also typically a disease of longevity, with the median age at diagnosis in the general population reported to be around 73 years [13]. The incidence of bladder cancer increases with age. In our evaluated cases, the median age at diagnosis was slightly younger at 55 years, and the oldest patient was 67 years. In the general (non-HIV infected) population in the United States bladder cancer is more prevalent in Caucasians than in African Americans and Hispanics. Our data is insufficient to comment on the racial demographics of HIV-associated bladder cancer.

Although the degree of immunosuppression has not proven to be a major risk factor in the development of several NADC, the duration of HIV infection appears to play an important role in tumorigenesis [1]. In our study, 91% of the patients were not markedly immunosuppressed at the time of diagnosis of their bladder malignancy. The median CD4+ cell count in our series was 280 cells/mm^3 ^and the average duration of HIV infection was 8 years prior to diagnosis of bladder cancer. Up to 55% of the patients in our study were on HAART, and one patient was on single agent zidovudine [3]. Two patients were started on antiretroviral therapy at the time of cancer treatment, one patient with HAART and one with zidovudine. Unfortunately compliance data was not available for many of these patients, but two were known to be noncompliant with HAART.

Interrupted antiretroviral therapy has certainly been linked to cancer development [14]. It is unclear if the degree of non-compliance on antiretrovirals was related to the bladder cancer in these patients. One patient (case 1), with a history of noncompliance with HAART, presented with hematuria after stopping his antiretrovirals for three months. Although several of the patients from the literature were diagnosed with HIV prior to the advent of HAART, none of the patients in this series suffered from significant HIV-associated co-morbidities, other than one individual with Kaposi Sarcoma and oral candidiasis. One patient, case 1, died of pulmonary Aspergillus infection. Also, one patient (case 5) did have a history of cervical cancer, but this was diagnosed decades prior to developing both HIV and bladder cancer. Most (80%) cases of urothelial carcinoma in our study were high grade at presentation. One individual developed carcinoma with neuroendocrine differentiation, which is a rare tumor with an aggressive behavior. We did not observe an obvious association between the degree of HIV-related immunosuppression and adverse response to therapy.

The major risk factors for transitional cell cancer of the bladder include tobacco use, exposure to chemicals (e.g. arylamines and cyclophosphamide with long-term use), and a history of pelvic radiation. Occupational risk factors include recurrent and early exposure to hair dye, and exposure to dye containing aniline, a chemical used in medical and industrial dyes. Other potential risk factors include chronic bladder inflammation (from recurrent urinary tract infections and/or urinary stones), consumption of *Aristolochia fangchi *(a herb used in some weight-loss formulas), a diet high in saturated fat, and a family history of bladder cancer. Several studies have indicated that the HIV-infected population uses tobacco at an increased rate as compared to the general population [15]. Over half the patients in our series consumed tobacco. Occupational or other chemical exposures were not identified this cohort. HIV-infected patients who develop non-Hodgkin lymphoma, are likely to be exposed to cyclophosphamide as part of their chemotherapy regimen. The risk of bladder cancer associated with cyclophosphamide is related to the total dose of the medication received and if the patient developed hemorrhagic cystitis. To date, there is no known clear association between antiretroviral medications and the development of bladder cancer. The treatment of cervical cancer, prostate cancer and certain lymphomas involving the pelvic lymph nodes, which can occur with greater frequency in the HIV population, may involve radiation of the pelvis. One of our patients had a history of cervical cancer treated in this manner, resulting in hemorrhagic cystitis and ultimately bladder cancer. Recurrent bacterial infections of the urinary tract and a history of *Schistosoma haematobium *infection may predispose patients to the development of SCC of the urinary bladder. Two of our patients suffered recurrent cystitis. However, both of these patients developed TCC. The clinical details about the patient with squamous cell carcinoma from the literature were not reported. A possible explanation for the younger age of onset of bladder cancer in our group of HIV-infected patients is earlier, or potentially more extensive, exposure to risk factors.

An interesting link between antiretroviral therapy and bladder cancer involves antagonists to the CXCR4 receptor. The CXCR4/CXCL12 axis appears crucial in the metastasis of bladder cancer. CXCR4 is a chemokine receptor present on human T-cell lymphocytes responsible for HIV entry into the host cell. CXCR4 has also recently been found to participate in the process of metastases of several human tumors, including bladder cancer. One study demonstrated high levels of CXCR4 expression in invasive and locally advanced bladder cancer tissue samples, low levels in superficial bladder tumor cells, and no expression in normal urothelial cells [16]. Exposure of bladder cancer cells expressing CXCR4 to CXCL12 (the cytokine known as stromal cell-derived factor-1) leads to cell migration and invasion of the extracellular matrix. Moreover, the addition of 4F-benzoyl-TE14011, a CXCR4 antagonist, to the environment reduces tumor cell invasion [17]. Currently, there are no approved CXCR4 antagonists for use in humans. However, based on the aforementioned published data the CXCR4/CXCL12 pathway is a promising target for the treatment of bladder cancer.

Initial symptoms of bladder cancer may include painless hematuria, infection, and urinary obstruction if the tumor is near ureteric orifices. Hematuria is the most common presenting symptom of bladder cancer, although its presence is nonspecific. Characteristically, hematuria in this setting is grossly apparent and may involve blood clots, is present throughout the urine stream, and is painless. All but one of the patients in our series with a recorded clinical history presented with hematuria. Constitutional symptoms including weight loss are infrequent, and typically associated with more advanced bladder cancer. Several of the patients reported symptoms of bladder irritation such as urinary frequency or urgency. Dysuria, and lower abdominal and pelvic pain were also present. Necrotic tissue within the bladder in several of cases may have become secondarily infected. The evaluation of any of the above signs and symptoms (dysuria, frequency, urgency and even hematuria) in patients infected with HIV involves a wide differential, which can lead to a delay in diagnosis of bladder cancer. One patient in our series initially presented with microscopic hematuria on routine urinalysis. Ultrasound at that time demonstrated bilateral nephrolithiasis and urine cytology was negative. One year later, he had gross hematuria and was diagnosed with bladder cancer. He had an aggressive tumor and ultimately died of metastatic bladder cancer.

Infection-related cystitis is probably the most common etiology for dysuria, hematuria, urinary urgency and frequency. Other causes to consider include stones (kidney and bladder), renal disease, medications (e.g., rifampin), urinary obstruction, trauma and in men over the age of 40 years benign prostatic hyperplasia (BPH). Immunosuppression and voiding dysfunction in HIV infected patients have been linked to an increased incidence of urinary tract infection in this population [18]. Sexually transmitted diseases, including gonorrhea and chlamydia, can cause dysuria, especially in men. In addition, acute bacterial prostatitis can present with dysuria and pelvic pain. An important consideration in the AIDS population is the potential for opportunistic and unusual infections. For example, *Toxoplasma gondii *can cause cystitis and even manifest as a bladder tumor in HIV-infected patients [19]. Also, several case reports have linked HIV infection with lower genitourinary tract condylomata from HPV infection [20]. Other opportunistic viral infections such as cytomegalovirus, adenovirus, and BK virus can all cause cystitis. BK virus specifically is an important cause of hemorrhagic cystitis and should be included in the differential of an HIV infected patient presenting with hematuria [21]. Lastly, *Mycobacterium tuberculosis *can lead to irritative bladder symptoms, usually as a consequence of the organism migrating from a primary kidney infection [22]. Metabolic derangements associated with HIV and its treatment can lead to dehydration and promote stone formation [18]. The use of protease inhibitors, specifically indinavir and recently atazanavir, is a well known cause of nephrolithiasis [23]. One of our patients (case 2) suffered from both recurrent bacterial cystitis and indinavir-related bladder stones. Finally, a malignancy at any location along the genitourinary tract can lead to hematuria, especially renal cell carcinoma. Several different forms of non-Hodgkin lymphoma have been reported involving the urinary tract. Primary T-cell lymphoma and B-cell lymphoma of the urethra have been diagnosed during the evaluation of hematuria in HIV-infected patients [24,25]. Rare cases of Burkitt lymphoma and anaplastic large cell lymphoma of the bladder have been reported in this population [26,27]. Given this extensive differential, hematuria in a patient with HIV infection should lead to prompt and thorough evaluation.

The diagnosis of bladder cancer includes urine cytology, urological tests (e.g. cystoscopy and biopsy) and imaging tests. Urine cytology and ultrasound were not helpful in identifying malignancy in our cohort. CT scan, particularly CT-urogram, did identify bladder wall thickening in several patients. As expected, cystoscopy was most effective at identifying lesions and allowed for biopsy and diagnosis at the same time. Staging, based on the depth of invasion through the bladder wall, is the principle factor in determining prognosis and management. Other prognostic factors identified in some series include hydronephrosis, anemia, expression of various proteins (e.g. blood group substances, epidermal growth factor receptor) and genetic abnormalities (e.g. aneuploidy, p53, Rb, p21). This molecular information was not available in any of these cases, but may be interesting to examine in future studies. The majority of our patients (7/9; 78%) presented at an early stage of bladder cancer without invasion of the muscularis propria, and only one patient had metastatic disease at diagnosis. This is consistent with the experience in the general population, in which 75% of cases are diagnosed with localized disease [13]. As initial, and potentially curative, therapy for early stage disease involves surgery, all patients in this study underwent either partial (TURBT) or complete resection of their tumors. After TURBT for superficial disease, approximately 40-80% patients will relapse and 10-25% of these cases will involve invasive or metastatic disease. Indeed, five patients in our series relapsed, three with superficial tumors and two with metastatic disease. A single patient had multiple relapses.

Intravesical immunotherapy or chemotherapy is standard after TURBT to prevent relapse in high risk patients. Additionally, the goals of adjuvant intravesical therapy include eradication of residual disease and prevention of disease progression. Several agents have been used in this capacity, however, immunotherapy with BCG has generally proven more efficacious than chemotherapy. BCG has a documented long-term progression free survival and bladder cancer-specific survival [28]. The goal of BCG administration is to create a local immune response which has anti-tumor effect. This local immune response includes the recruitment and activity of CD4+ T-helper cells. Intravesical chemotherapy with mitomycin C is also effective in the adjuvant setting. Three patients in our series received intravesical mitomycin C or epirubicin following TURBT. Two of these patients recurred and required cystectomy, and one recurred with metastatic disease and died.

As BCG is a live, attenuated strain of *Mycobacterium bovis*, it is not typically used in immunosuppressed (including HIV-infected) patients as there is a theoretical increased risk of life-threatening disseminated infection. We are aware of one case report that documented the development of bilateral interstitial pneumonitis in an HIV-infected patient after intravesical therapy with BCG [29]. Systemic *M. bovis *infection after intravesical administration is a rare occurrence in the general population, and is typically responsive to antituberculosis therapy. One study presented the uncomplicated use of intravesical BCG in three renal transplant recipients. In that study, two patients received prophylactic antituberculosis therapy concurrently with their intravesical infusions, and none developed systemic *M. bovis *infection [30]. Anecdotally, intravesical BCG has been used safely in HIV infected patients with bladder cancer. A single patient in our series received an infusion of intravesical BCG in an effort to prevent cystectomy, and he did not develop infectious complications. In addition to the infection concerns, intravesical BCG may be ineffective in patients with HIV. Intravesical BCG is dependent on a functioning immune system to exert its local effects. Due to the decrease in CD4+ cells, BCG may not be able to sufficiently stimulate the host immune system to create the necessary anti-tumor activity in the HIV-infected patient. Given the risk of disseminated infection and potential suboptimal response to this therapy in HIV-infected patients, BCG therapy should probably be used with caution or avoided until further studies are performed. The inability to use intravesical BCG in the management in bladder cancer, however, underscores the importance of early detection in this population.

Invasion of the muscularis propria is an indication for radical cystectomy, as partial resection in this group of individuals may lead to inferior results. A 2003 trial demonstrated a survival benefit to neoadjuvant chemotherapy for patients with muscle-invasive disease. The two patients in this study who presented with muscle-invasive disease received adjuvant cisplatin-based chemotherapy, but both died within 6 months of diagnosis. Two additional patients developed metastatic disease and received metastasis-specific treatment for palliation (whole brain and bone radiation) without systemic chemotherapy and died. In the Surveillance Epidemiology and End Result (SEER) data for bladder cancer, the 5-year survival for localized, regional and distant disease is 92.5%, 44.7% and 6.1% respectively [13]. Our findings are consistent with this data in that patients who had only superficial disease, even with relapse, did well with resection and local therapy, and those with regional or metastatic disease did poorly.

## Conclusion

We present the largest series of HIV-associated bladder cancer involving 11 patients. Bladder cancer is part of the growing list of cancers likely to be encountered in HIV-infected patients now living longer with controlled HIV disease. Acknowledging the limitation of our small retrospective study with incomplete information in some of the cases, these data suggest that such HIV-positive patients with bladder cancer are likely to present at a slightly younger age and with only mild immunosuppression. Hematuria, dysuria, frequency and/or urgency in an HIV-infected patient warrants complete evaluation, and the differential diagnosis should include bladder cancer. Persistent urinary symptoms should be evaluated by a specialist and special investigations including cystoscopy. Most patients in our series presented with early stage disease, and despite their frequent relapses and development of subsequent metastases, followed an expected course for their bladder cancer. HIV-positive cigarette smokers and those who chew tobacco should be counseled on the potential risk of bladder malignancies. Finally, further observation and investigation are needed to better understand the natural history and appropriate treatment of bladder cancer in the HIV-infected population.

## Competing interests

The authors declare that they have no competing interests.

## Authors' contributions

BD, MB, DA, GB, TC and LP identified patient cases and assisted in the acquisition of data. All authors assisted in the analysis and interpretation of data. EG, BD and LP prepared the initial manuscript. All authors revised the manuscript and all have read and approve of the final manuscript.

## Pre-publication history

The pre-publication history for this paper can be accessed here:


